# The complete chloroplast genome of *Schisandra repanda* (Siebold & Zucc.) Radlk. (Schisandraceae)

**DOI:** 10.1080/23802359.2026.2620178

**Published:** 2026-01-28

**Authors:** Mi Sun Lee, Minjee Lee, Namsu Jo, Hee Jeong Jeong, Chang Pyo Hong, Sang-Ho Kang, Soo Jin Kwon, Chang Kug Kim, Ho Bang Kim, Jungho Lee, Yi Lee

**Affiliations:** a Department of Industrial Plant Science & Technology, Chungbuk National University, Cheongju, Republic of Korea; b Green Plant Institute, Yongin, Republic of Korea; c Biotechnology Research Institute, Jenong S&T Co., Ltd, Anseong, Republic of Korea; d Department of Crop Science and Biotechnology, General Graduate School, Dankook University, Cheonan, South Korea; e Genomics Division, National Institute of Agricultural Sciences (NAS), Jeonju, Republic of Korea; f Department of Environmental Horticulture, School of Equine Science and Horticulture, Cheju Halla University, Jeju, Republic of Korea

**Keywords:** *Kadsura*, *Illicium*, medicinal plant, phylogenetic analysis

## Abstract

The complete chloroplast genome of *Schisandra repanda* (Siebold & Zucc.) Radlk., 1886, was assembled and annotated for the first time using NGS data. The genome is 146,620 bp in length and consists of a large single-copy (LSC) region of 95,338 bp, a small single-copy (SSC) region of 18,330 bp, and two inverted-repeat (IR) regions of 16,476 bp each. A total of 125 genes were annotated, including 82 protein-coding genes, 8 rRNA genes, and 35 tRNA genes. These results provide fundamental genomic information for species identification, comparative studies of *Schisandra*, and climate change response relevant to conservation.

## Introduction

*Schisandra repanda* (Schisandraceae) is a deciduous broad-leaved creeper occurring in Korea and Japan, along with the genera *Schisandra*, *Illicium*, and *Kadsura* (Kitamura and Okamoto 1959; Suh 2007). In Korea, *S. repanda* distributes from 600 to 1400 m above sea level on Mt. Halla of Jeju Island (Lee et al. 1999, 2001). The species is distinguished from *S. chinensis* by its black, rather than red, berries. The fruit of *S. repanda* is classified as a high-moisture fruit (85%–87%) (Shin et al. 1998). It is utilized for its medicinal properties, including anticancer, antioxidant, and anti-inflammatory properties (Li et al. 2018; Zhou et al. 2021). The species, once abundant in the wild of Korea and Japan until about 20 years ago, has now become scarce due to habitat loss from climate change (Kim et al. 2010). The chloroplast genomic information of *S. repanda* is not available, though the mitochondrial genome was recently published (Lee et al. 2023a, 2023b). In our study, the complete chloroplast genome of *S. repanda* was assembled, and the result would enhance our understanding of the genetic diversity of Schisandraceae and aid the conservation of *S. repanda*.

## Materials and methods

### Plant sampling

**Figure 1.**
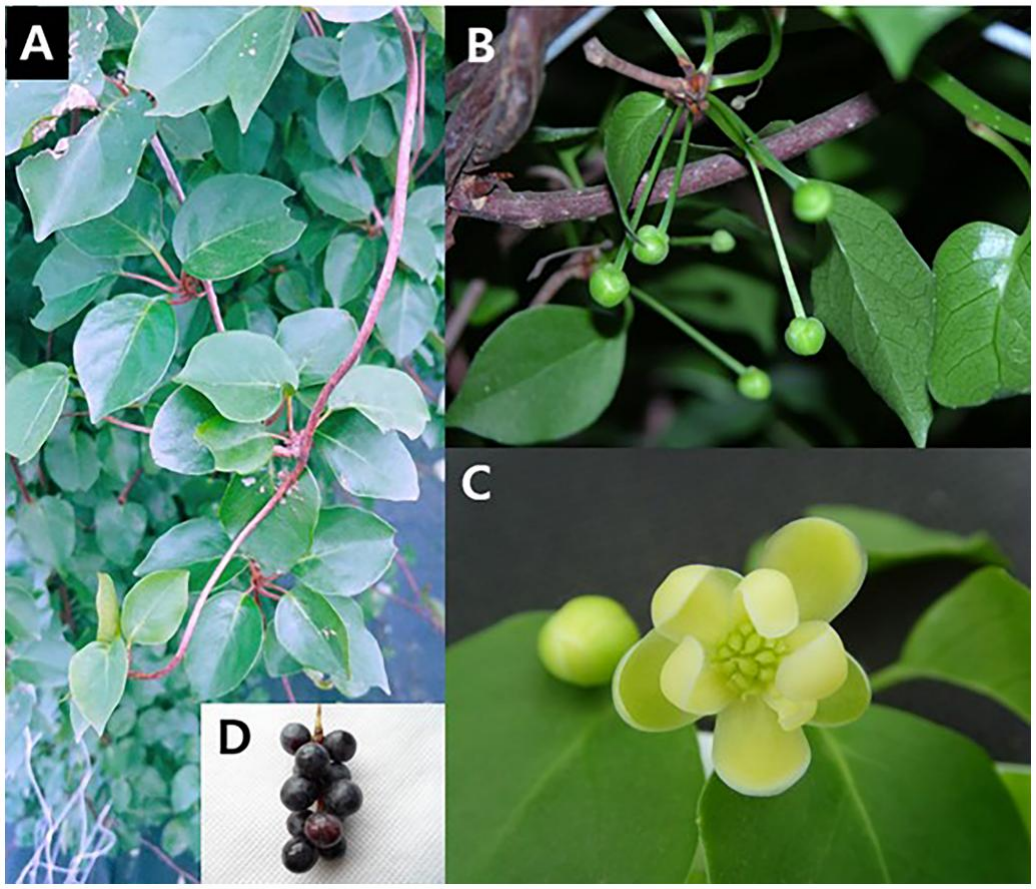
The female plant of *S. repanda* at different stages. A: Vegetative stage showing deciduous broad-leaved creeper. B. Female floral bud at the branches. C. Bloomed female flower. D: fruit stage showing blackberries. The photo was taken by Yi Lee in Jeju-Special Self-Governing Province Agricultural Research & Extension Services (33° 15′ 39.9ʺ N, 126° 29’ 20.2ʺ E).

Leaf samples of female plant were collected from Jeju-Special Self-Governing Province Agricultural Research & Extension Services (33° 15′ 39.9ʺ N, 126° 29′ 20.2ʺ E), Korea (Figure 1). A dried voucher specimen was deposited in the Herbarium of the National Institute of Horticulture and Herbal Science, Eumsung, Korea (IN; http://www.nihhs.go.kr/; voucher number: MPS006517-2; contact: Yoongee Lee, yoong0625@korea.kr).

### DNA isolation, sequencing, assembly, and annotation of the chloroplast genome

DNA was extracted with DNeasy Plant Mini Kit (Qiagen, Valencia, CA, USA) following the manufacturer’s instructions. Leaf samples and DNA were stored at −70 °C. A genomic library was constructed using the Illumina HiSeq platform. Raw data were trimmed using the CLC quality trim program (ver. 4.21) in the CLC Assembly Cell package (version 4.2.1, https://www.qiagenbioinformatics.com/products/clc-assembly-cell/) (Jeong et al. 2014). Trimmed reads were assembled with the dnaLCW method in the CLC *de novo* assembler under default parameters, and contigs below the cutoff length were discarded. Chloroplast-derived contigs were identified, extended, merged, and gap-filled. Loci were annotated with GeSeq (https://chlorobox.mpimp-golm.mpg.de/geseq-app.html), (Tillich et al. 2017). The complete chloroplast genome of *S. repanda* was registered with the NCBI as sequence accession no. OK377339.

### Phylogenetic analysis

We performed phylogenetic analysis using the complete chloroplast genome sequence of *S. repanda* with 20 species, including 15 ingroup species of Schisandraceae and five outgroup species of the Nymphaeeales, based on Qiu et al. (1999) and Lee et al. (2023a, 2023b). Chloroplast genome sequences of the other 20 species were obtained from NCBI. The tree was rooted with representatives of *Kadsura* and *Illicium*. Outgroup species included *Nuphar shimadae* MH050797, *Brasenia schreberi* MZ328718, *Cabomba aquatica* MG720559 (Gruenstaeudl et al. 2018), *Euryale ferox* MH778542, *Nymphaea gigantea* MT107637 (Sun et al. 2021). A phylogenetic tree was constructed in MEGA-X using the maximum-likelihood method (Nei and Kumar 2000; Hong et al. 2017; Kumar et al. 2018) based on 18 chloroplast genes (*accD, atpA, atpB, matK, ndhD, ndhF, ndhH, psaA, psaB, psbA, psbB, psbC, rbcL, rpoB, rpoc1, rpoC2, ycf1,* and *ycf2*) from 21 taxa. The tree was generated under the time-reversal model of the maximum likelihood with 1000 bootstrap replications.

## Results

**Figure 2.**
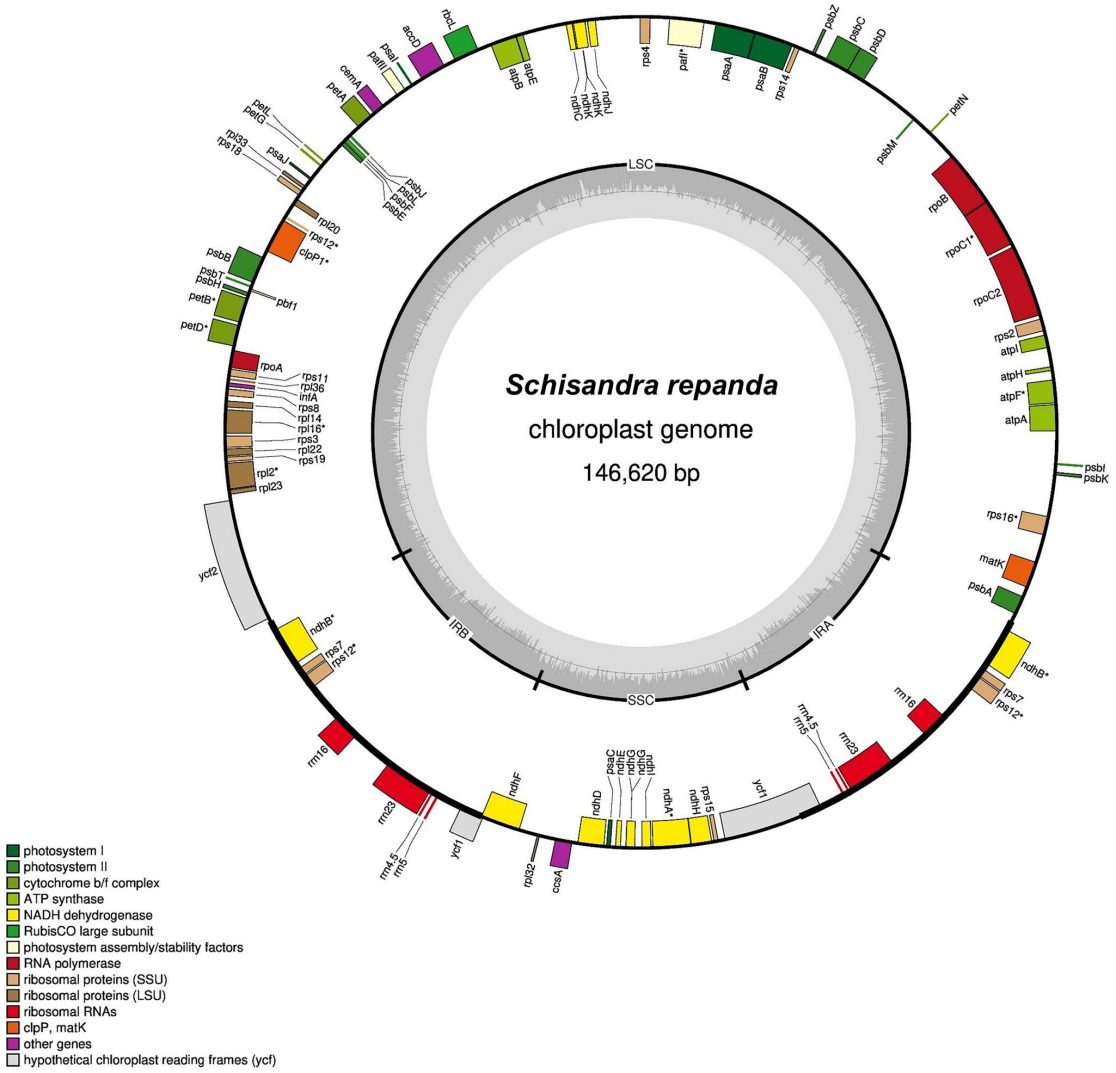
Chloroplast genome map of *S. repanda*. The map was prepared using the GeSeq program (https://chlorobox.mpimp-golm.mpg.de/geseq-app.html), (Tillich et al. 2017). GC contents are indicated on the inner circle. Genes that are transcribed are indicated on the outside and inside of the large circle, respectively.

*S. repanda* chloroplast genome was 146,620 bp, comprised of a large single-copy (LSC, 95,338 bp), a small single-copy (SSC, 18,330 bp), and two inverted-repeat regions (IRs, 16,476 bp each) (Figure 2). The GC content was 39.66%, varying across LSC, SSC, and IR. The chloroplast genome contained 125 genes: 82 protein-coding, 8 rRNA, and 35 tRNA. Four genes *(ndhB*, *rps7*, *rps12*, and *ycf1*) had two copies; rRNA (*rrn16, rrn23, rrn4.5*, and *rrn5*) and tRNA (*trnA_UGC*, *trnI_GAU*, *trnN_GUU*, *trnR_ACG*, *trnV_GAC*) were also duplicated. Read coverage was relatively uniform, averaging 958× (Figure S1). One ycf1 was a pseudogene, the other a hypothetical chloroplast RF1. Twelve genes carried introns: *rps16, atpF, rpoC1, ycf3, clpP, petB, petD, rpl16, rpl2, ndhB* (two copies), and *ndhA* (Figure S2). *rps12* was *trans*-spliced, with exon 1 in LSC and two copies of exons 2 and 3 in IR (Figure S3).

**Figure 3.**
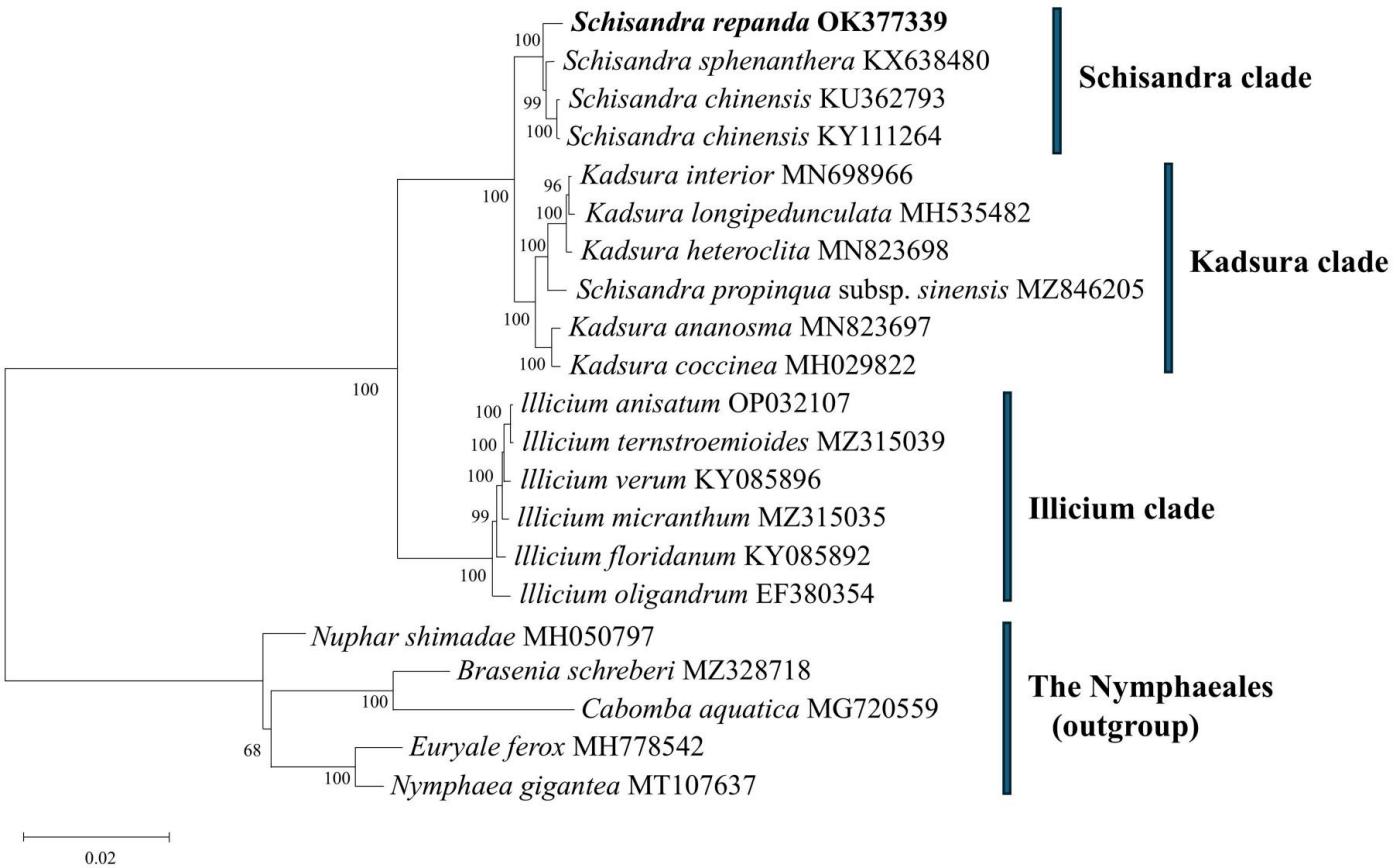
Maximum-likelihood phylogenetic tree based on 18 chloroplast genes (*accD, atpA, atpB, matK, ndhD, ndhF, ndhH, psaA, psaB, psbA, psbB, psbC, rbcL, rpoB, rpoc1, rpoC2, ycf1, and ycf2*) sequences of *S. repanda* and 15 species belonging to the Schisandraceae family. Numbers at nodes represent bootstrap values for 1,000 replicates. The following 20 sequences were used: *S. sphenanthera* KX638480, *S. chinensis* KU362793, *S. chinensis* KY111264, *Kadsura interior* MN698966 (Fu et al. 2020), *Kadsura longipedunculata* MH535482, *Kadsura heteroclita* MN823698 (Wang et al. 2020), *Schisandra propinqua* subsp. *sinensis* MZ846205, *Kadsura ananosma* MN823697 (Liu et al. 2020), *Kadsura coccinea* MH029822 (Li and Zheng 2018), *Illicium anisatum* OP032107, *Illicium ternstroemioides* MZ315039, *Illicium verum* KY085896, *Illicium micranthum* MZ315035, *Illicium floridanum* KY085892, *Illicium oligandrum* EF380354 (Hansen et al. 2007), *Nuphar shimadae* MH050797, *Brasenia schreberi* MZ328718, *Cabomba aquatica* MG720559 (Gruenstaeudl et al. 2018), *Euryale ferox* MH778542, *Nymphaea gigantea* MT107637 (Sun et al. 2021).

Phylogenetic analysis clustered *S. repanda* with 15 taxa of Schisandraceae into three clades. All six *Illicium* species formed a basal clade. Two additional clades were identified. Four *Schisandra* taxa formed one clade, with *S. rapenda* at its base. The other clade contained four *Kadsura* and one *Schisandra* species, here termed the *Schisandra* and *Kadsura* clade (Figure 3). Interestingly, *Schisandra propinqua* subsp. *sinensis* (MZ846205) was placed in the *Kadsura* clade. *S. repanda* was placed at the base of the *Schisandra* clade, and its closest taxon was *Schisandra sphenanthera*.

## Discussion and conclusion

Considering the limited research on the chloroplasts of *S. repanda*, the complete chloroplast genome assembly strengthens research on its conservation and phylogenetic relationships. In this study, the chloroplast genome sequence of *S. repanda* was assembled and annotated. Phylogenetic analysis using complete chloroplast genomes positioned *S. repanda* within the *Schisandra* clade, closely related to *S. chinensis* and *S. sphenanthera*, supporting the taxonomic classification within the Schisandraceae (Figure 3). The chloroplast genome of *S. repanda* (146,620 bp; GC 39.7%) is slightly smaller than that of *S. chinensis* (147,772 bp and 146,859 bp) and comparable to *S. sphenanthera* (146,843 bp). GC content is nearly identical across taxa (39.5%–39.7%). A total of 126 genes were annotated in *S. repanda*, comparable to *S. chinensis* (125 and 126) and *S. sphenanthera* (116), indicating a conserved plastome architecture in *Schisandra*.

We compared five partial mitochondrial sequences of *S. repanda* (OK077167-OK077171) with its chloroplast genome (OK377339). The mitogenome contained 59 genes, of which 23 were shared. Both genomes retained conserved a rRNA (*rrn5*) and 12 tRNAs (*trnC-GCA, trnD-GUC, trnE-UUC*, *trnF-GAA, trnM-CAU*, *trnN-GUU, trnP-UGG, trnR-UCU, trnS-GCU, trnT-GGU, trnW-CCA*, and *trnY-GUA)* essential for translation. This shared set reflects co-evolutionary constraints, as organellar RNAs must interact with nuclear-encoded ribosomal proteins (Sloan et al. 2018).

Gene duplications in the IR regions may stabilize the plastome and balance gene dosage (Jansen and Ruhlman 2012; Krämer et al. 2024). Among the two *ycf1* copies, one was annotated as a pseudogene, the other (≈5.7 kb) retains an intact ORF as a hypothetical chloroplast RF1, consistent with reports that *ycf1* occurs as both functional and pseudogene in angiosperm (Dong et al. 2015; Liu et al. 2018).

*S. repanda* thrives at 600–1400 m, indicating adaptation to this range. Its chloroplast genome contains genes potentially linked to environmental adaptation. In particular, *accD*, *ndhD*, *ndhF*, and *ndhH* function in photosynthetic electron transport and energy conversion, which may maintain photosynthetic efficiency under stress. Additionally, *rbcL* and *psaA* are vital for carbon fixation and photosystem function, supporting photosynthesis under stress. Given its restricted habitat, comparative genomic studies across altitudes could help elucidate mechanisms of adaptation.

In addition, Jeong et al. (2021) analyzed chloroplast sequences of *S. chinensis*, *S. repanda*, and *Kadsura japonica*, developing four InDel markers to prevent misidentification and adulteration. Using the same samples, the new *S. repanda* chloroplast genome allows detailed characterization of variation and marker structures. These findings support marker validation and polymorphism analysis, improving classification and phylogenetic studies, and provide a basis for precise identification and quality control of related herbal materials.

## Ethics approval and consent to participate

*S. repanda* samples were collected by Yi Lee with the permission of Seok Chul Yang, researcher of Jeju-Special Self-Governing Province Agricultural Research & Extension Services. Jungho Lee identified the voucher specimen (voucher number: MPS006517-2). The authors comply with relevant institutional, national, and international guidelines and legislation for plant study.

## Supplementary Material

Figure S2_600dpi.jpg

Figure S3_600dpi.jpg

Figure S1_600dpi.jpg

## Data Availability

The genome sequence data that support the findings of this study are openly available in GenBank of NCBI (https://www.ncbi.nlm.nih.gov/) under accession no. OK377339. The associated “BioProject,” “SRA,” and “Bio-Sample” numbers are PRJNA1137126, SRR29876393, and SAMN42568013, respectively.
