# Supplementary figures and images for: The complete chloroplast genome of Schisandra repanda (Siebold & Zucc.) Radlk. (Schisandraceae)
Source: Mitochondrial DNA B Resour. 2026 Jan 28;11(2):312–6. doi: 10.1080/23802359.2026.2620178 (PMC12857660; doi:10.1080/23802359.2026.2620178)

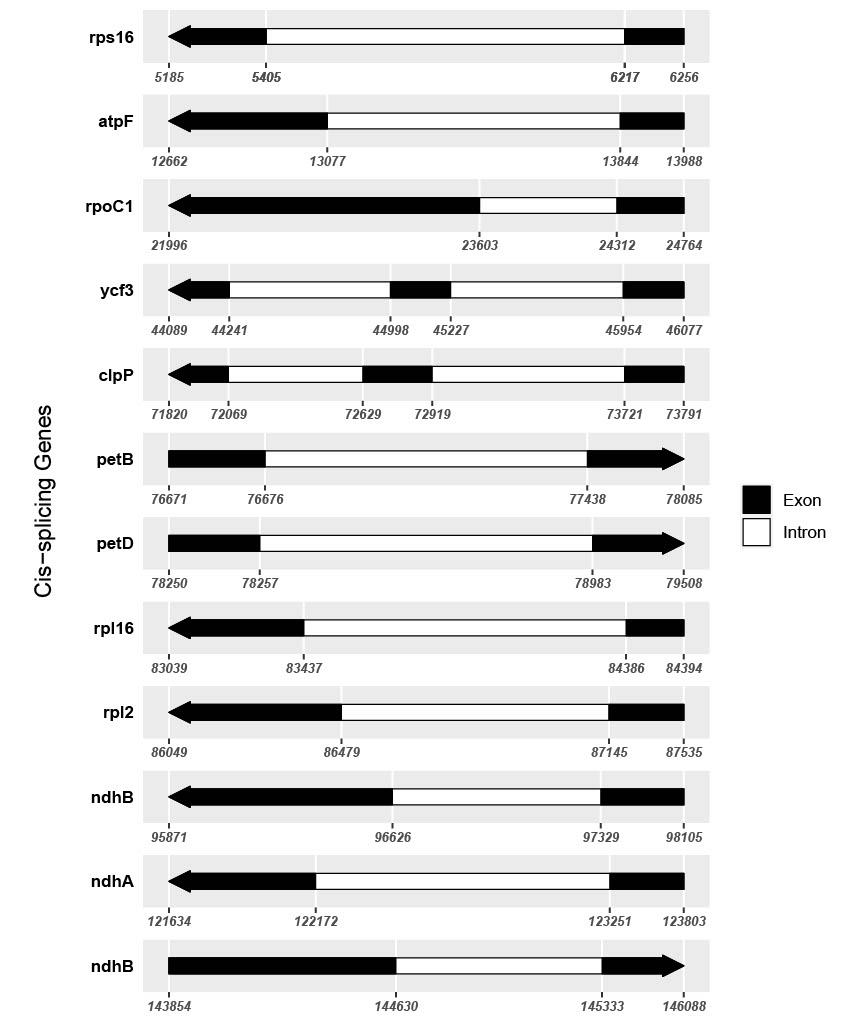

Supplement: Figure S2_600dpi.jpg [file TMDN_A_2620178_SM5864.jpg]

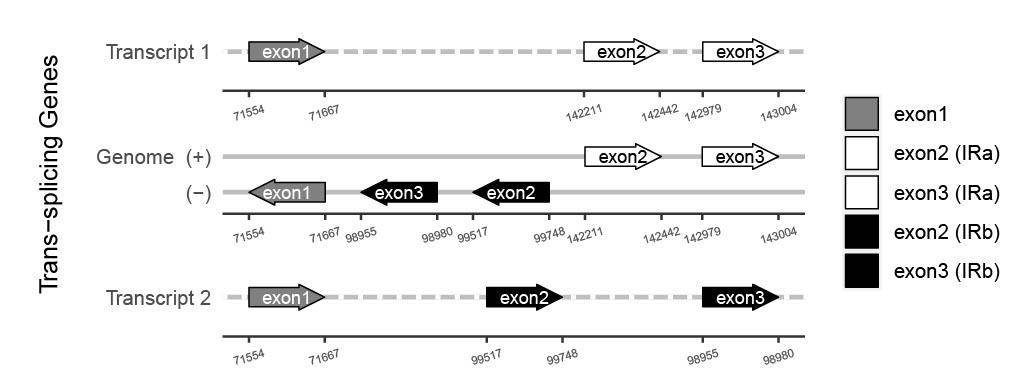

Supplement: Figure S3_600dpi.jpg [file TMDN_A_2620178_SM5863.jpg]

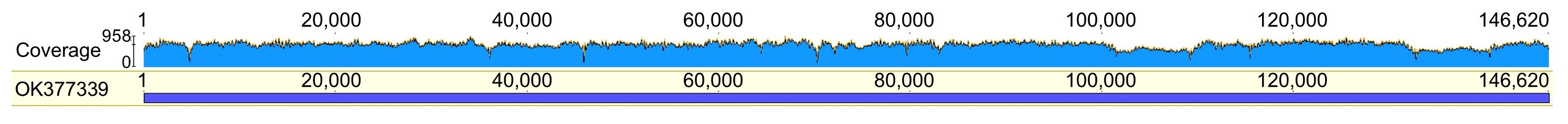

Supplement: Figure S1_600dpi.jpg [file TMDN_A_2620178_SM5862.jpg]
